# Nasal Bone Fracture (NBF): A Retrospective Study on Epidemiology and Treatment Outcomes in the Omani Population

**DOI:** 10.7759/cureus.73725

**Published:** 2024-11-15

**Authors:** Bassel Adra, Salma Al Sheibani

**Affiliations:** 1 Otolaryngology Head and Neck Surgery, Aintree University Hospital, Liverpool, GBR; 2 Ear, Nose and Throat (ENT) Head and Neck Surgery, Al Nahdha Hospital, Muscat, OMN

**Keywords:** epidemiology, nasal bone fractures, omani population, septorhinoplasty, sports injuries

## Abstract

Purpose

To investigate the epidemiology, etiology, treatment outcomes, and sequelae of nasal bone fractures (NBFs) in the Omani population, which may contribute to developing better treatment approaches and preventive strategies.

Materials and methods

A retrospective chart review was conducted at Al Nahdha Hospital from January 2012 to January 2017. Data on demographics, injury mechanisms, treatment modalities, and outcomes were collected for 453 patients with NBFs. All patients with fresh NBFs, children, or adults of both genders, were included. Of these, 171 patients underwent nasal bone reduction under general anesthesia (GA). The collected data included the patients' age, gender, cause of injury, treatment, and surgical outcome. SPSS was used for data collection and analysis.

Results

Sports-related injuries, particularly football, were the most common cause (35.97%), followed by accidental falls (32.92%), road traffic accidents (17.68%), and assaults (12.82%). Most cases (98.8%) were managed with closed reduction, with a satisfactory outcome in 85.81%. However, 14.18% experienced residual deformities requiring further surgery. The highest frequency of displaced NBF was in the 21-30 age group, with males being the predominant patients.

Conclusion

NBFs are particularly common among young males (21-30 years). Sports injuries, falls, and road traffic accidents are the primary causes. Timely and appropriate management is crucial to minimize long-term complications. Hence, an emphasis on health education and preventive measures for sport injuries among the young population is required.

## Introduction

Nasal bone fractures (NBFs) are among the most common facial bone injuries, constituting approximately 50% of all facial fractures and ranking as the third most frequent fracture in the human skeleton [1-2]. Given its prominent position, the nose is particularly susceptible to trauma, often resulting in fractures that may involve both bony and cartilaginous structures, including the nasal septum and upper lateral cartilages. The high prevalence of concomitant nasal septal injuries, estimated at up to 90% of cases, further underscores the complexity of these fractures.

While the etiology of NBFs is diverse and influenced by various factors, including geographic location, socioeconomic status, and cultural practices, limited epidemiological data exist, particularly from Middle Eastern regions.

This retrospective study, conducted in the Department of Otolaryngology at Al Nahdha Hospital, a tertiary care teaching hospital in Muscat, Oman, aims to provide a detailed analysis of the epidemiology and etiology of NBFs across different age groups, assess optimal management approaches, and document post-treatment outcomes.

## Materials and methods

A retrospective review was conducted at Al Nahdha Hospital in Muscat, Oman, from January 2012 to January 2017. The study was approved by the hospital's ethics committee. The study aimed to investigate the epidemiology, etiological factors, management strategies, and outcomes of NBFs over a five-year period.

Data collection

The medical records of patients presenting to the emergency department and ENT clinic with NBFs were reviewed. The data collected from each patient's medical record included demographics (age and gender), mechanism of injury (sports injuries, accidental falls, road traffic accidents (RTAs), physical assaults, and cases with unspecified causes), treatment provided (closed reduction, open reduction, or conservative management), and the outcome (patient satisfaction, functional outcomes, and need for further surgery).

Inclusion and exclusion criteria

Patients with displaced NBFs confirmed by clinical and radiological assessment were included in the study, while patients with incomplete medical records or those who did not undergo any treatment were excluded.

Statistical analysis

Descriptive statistics were used to summarize the demographic data, injury mechanisms, and treatment outcomes. These included demographics and injury characteristics, comparison of treatment outcomes, and follow-up analysis.

## Results

Demographics

The retrospective analysis comprised a total of 171 cases of displaced NBFs requiring reduction. Among these patients, 140 were male (81.87%) and 31 were female (18.13%), reflecting a significant male predominance. The age of the patients ranged from 2 to 55 years, with a mean age of 26.5 years.

The distribution of NBFs by age group showed that the highest incident rate was in the 21-30 years group, 38.01% (n=65), then 13-20 years, 25.73% (n=44), followed by 2-12 years, 19.88% (n=34), 31-40 years, 13.45% (n=23), and finally 41-60 years, 2.92% (n=5).

This analysis indicates that the highest incidence of NBFs occurred in the 21-30-year age group, followed by the adolescent group (13-20 years).

Overall, a significant male-to-female ratio of 4.5:1 was observed across all age groups, suggesting a higher susceptibility to NBFs among males in this population (Table 1).

**Table 1:** Distribution according to age and gender.

Gender/Age	0-12 years	13-20 years	21-30 years	31-40 years	41-60 years	All cases	% According to gender
Males	20	41	58	17	4	140	81.87%
Females	14	3	7	6	1	31	18.13%
All cases	34	44	65	23	5	171	100%
% according to age	19.88%	25.73%	38.01%	13.45%	2.92%	-	

Etiology

The analysis identified four primary causes of NBFs in the studied population. The first was sports injuries, which emerged as the leading cause of NBFs, accounting for 59 cases (34.50%). Within this category, football injuries were particularly prevalent. These injuries commonly resulted from player collisions, often occurring during actions such as heading the ball or when another player’s elbow struck the lateral wall of the nose. The nature of these injuries frequently led to displaced fractures and deformities, which were often accompanied by fractures or dislocations of the septal cartilage.

**Figure 1:**
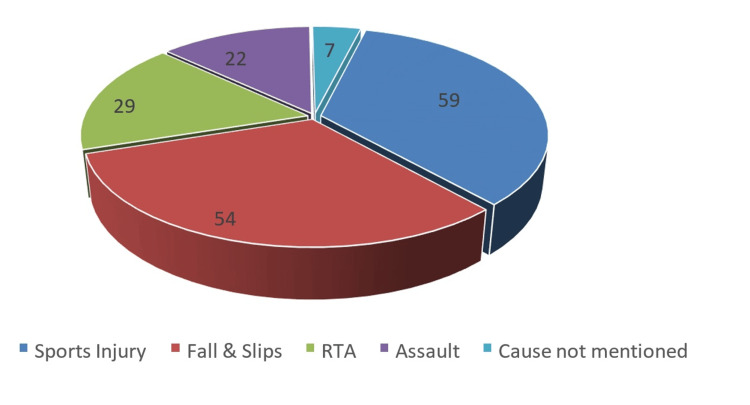
Causes of nasal trauma (n=171).

Accidental falls constituted the second most common cause of nasal fractures, with 54 cases recorded (31.58%). This cause was especially significant among pediatric patients, where falls typically resulted from impacts with doors, walls, or other objects. The third common cause was road traffic accidents (RTAs), which accounted for 29 cases (16.96%) of nasal fractures. These injuries often involved significant trauma and, in some cases, resulted in complex fractures requiring specialized management. Finally, physical assaults were identified as the cause in 22 cases (12.87%). The motivations for these assaults may vary but can be attributed to interpersonal conflicts or altercations. However, in seven cases (4.09%), the cause of the nasal fracture was not documented (Figure 1).

Age and trauma distribution

**Figure 2:**
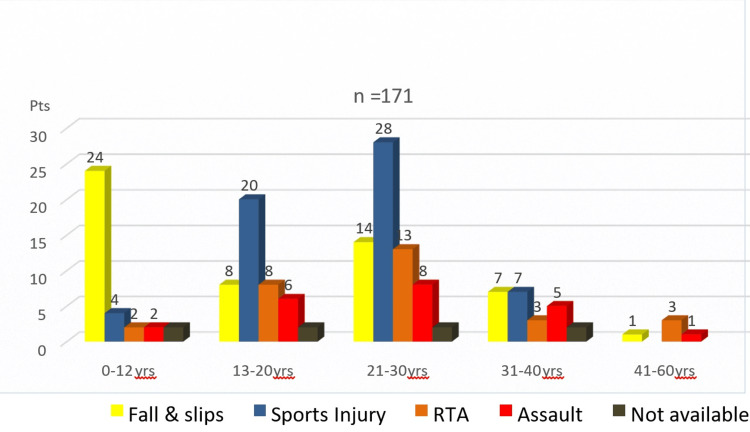
The distribution of etiology in relation to the age group.

When examining the distribution of trauma causes by age group, several patterns emerged. The pediatric group showed that falls were the predominant cause of nasal fractures, accounting for 24 out of 34 cases (70.58%), while the adolescent and young adult group revealed that sports injuries, primarily from football, were the most common among patients aged 13-30 years, accounting for 66 cases (43% of the total). In the young adult group, RTAs were particularly prevalent in the 20-30-year age group, indicating a trend that warrants further investigation (Figure 2).

The management approach for most cases (n = 169, 98.8%) involved the closed reduction of NBFs and manipulation of the septal cartilage, conducted within two weeks of the trauma. However, two cases involving comminuted fractures resulting from RTAs required open reduction, necessitating collaboration with the oral and maxillofacial surgery team (Table 2).

**Table 2:** Reduction type.

Reduction type	Number	Percentage
Closed reduction	169	98.8%
Open reduction	2	1.2%

Surgical outcomes

Postoperative follow-up evaluations were conducted at two-week, three-month, and six-month intervals in the septorhinoplasty clinic. The assessments focused on nasal patency, functional improvement, nasal shape, and patient satisfaction.

Among the 171 patients, 148 who completed the follow-up, 127 (85.81%) expressed satisfaction with their functional and aesthetic outcomes, while 21 patients (14.18%) reported dissatisfaction, primarily due to residual septal or external deformities. However, 23 patients (13.45%) lost their follow-up (Table 3).

**Table 3:** Residual septal and external deformities requiring surgery. SRP: Septorhinoplasty.

Revision surgery	Number	Percentage
Needed SRP	19	12.83%
Needed Septoplasty	2	1.35%

Of these dissatisfied patients, 12.83% (n=19) required further corrective septorhinoplasty, while two patients underwent septoplasty alone. Notably, all 19 cases necessitating further surgery were attributed to sports-related nasal traumas, specifically those related to football.

Pediatric nasal trauma

In the pediatric subgroup, accidental falls were responsible for 24 out of 34 cases (70.58%), leading to depressed fractures of the nasal dorsum or lateral nasal walls. Additionally, two cases resulting from RTAs were associated with severe maxillofacial injuries and required open reduction.

Successful outcomes were reported in 94.2% (n=32) of the pediatric cases managed with closed reduction. However, 5.8% (n=2) required subsequent septorhinoplasty due to persistent breathing difficulties and external deformities.

## Discussion

This study provides a detailed analysis of NBFs in Oman, offering insight into the epidemiology, etiology, and outcomes of this common facial injury. The predominance of sports-related injuries, particularly among young males, reflects both the demographic characteristics of Oman’s population and the cultural significance of football in the region. Compared to other studies from the Middle East and globally, our findings underscore some unique patterns while confirming general trends regarding nasal fractures.

In line with findings from Ashoor et al. in Saudi Arabia and Zandi M et al. in Iran, this study demonstrates that young males are disproportionately affected by NBFs [3, 4, 5]. However, while both of these studies identified RTAs as the leading cause of nasal fractures, our study found that sports-related injuries, particularly football, were the most common cause (34.50%, n=59). This is likely due to the popularity of football in Oman and the Middle East, where the sport is played extensively at both amateur and professional levels.

Interestingly, global studies from East Asia, South America, and Europe report assault injuries as the primary cause of nasal fractures, often attributed to alcohol or drug abuse in the regions [6]. The high prevalence of nasal fractures among young males aged 13-30 years can be attributed to their increased participation in sports activities, particularly football, where physical contact and collisions are frequent.

Accidental falls, particularly in children aged 2-12 years, accounted for 31.58% (n=54) of fractures. This finding is consistent with other studies showing that young children are prone to falls, often due to developmental factors, lack of coordination, or environmental hazards (e.g., uneven surfaces or open doors). However, this is in sharp contrast to the United States, where automobile accidents (40%) are most common, followed by sports (25%) and intended home injuries (10%), as reported [7]. In Japan, Yabe T et al. [8] cites sports injuries as the most common cause of NBFs in pediatric age groups.

Accurate history and clinical examination with findings of crepitus provide a lot of information. As reported in many studies, except for medico-legal cases, a plain X-ray of nasal bones has no role in management [9-13]. It carries high false negative and false positive rates due to the presence of suture lines, old fractures, vascular markings, and soft tissue swelling that may hide the thin fracture lines [13-15]. Instead, the use of ultrasound and CT scans is increasingly being used in the diagnosis and management of fractured nasal bones.

In children, underdeveloped nasal bones and soft, compliant cartilages bend easily with blunt trauma, resulting in the dislocation or distortion of septal cartilage. Soft tissue edema is more generalized, and septal hematoma is proportionally more common in pediatrics than adults [16].

The management of NBFs has been controversial and challenging over the centuries since its description by Hippocrates [17]. In our series, 85.81% of operated patients were satisfied with the closed reduction of fractured nasal bones and associated septal fractures, while post-reduction deformities requiring septorhinoplasty or septoplasty made up 14.1% (n=21). This compares with published post-reduction failure rates of 22.5% by Basheeth N et al., while Dickson MG, Sharpe DT found a rate of 14-50% in their series [18].

One cause of reduction failure is attributed to an uncorrected septal fracture causing the nasal bone to heal in the direction of the deviated and fractured septum [19, 20]. Accordingly, managing a septal fracture is considered key to the successful reduction of fractured nasal bones. In our experience, proper manipulation and straightening of a deviated or fractured septum during the routine closed reduction of fractured nasal bones provides an equally satisfactory result.

While closed reduction remains the preferred treatment for NBFs due to its minimally invasive nature, it is not without limitations. In cases where there is significant cartilage involvement or multiple fracture lines, the outcomes may be suboptimal, requiring secondary interventions such as septorhinoplasty.

However, the study is not without its limitations. First, the retrospective design inherently carries the risk of incomplete or missing data. In our study, 23 patients were lost to follow-up, which may have affected the accuracy of the outcome assessments. Additionally, while the study focused on a single tertiary care hospital in Muscat, the findings may not be fully generalizable to other regions of Oman, where demographics and causes of nasal fractures could differ.

Another limitation is the reliance on closed reduction as the primary treatment method. While this is a common approach for nasal fractures, the study did not explore in detail the potential benefits of alternative or adjunctive treatments, such as the use of external nasal splints, which may yield better outcomes in certain cases.

Further prospective studies are needed to evaluate the long-term outcomes of different treatment modalities for NBFs. Additionally, research focusing on the use of advanced imaging techniques, such as 3D CT scans, may improve the precision of fracture assessments and optimize surgical outcomes. Future studies could also explore the impact of protective gear in reducing the incidence and severity of sports-related nasal injuries.

## Conclusions

The study indicates that sports injuries, particularly among the 21-30 age group, are a significant cause of NBFs in the Omani population. This highlights the need for health education and preventive measures targeting sports-related injuries in young people, such as wearing protective headgear during contact sports. Additionally, coaches and sports organizations should be encouraged to enforce safety protocols to reduce the risk of nasal and facial injuries.

Optimal management of NBFs in the acute phase is essential to prevent secondary functional and aesthetic deformities. The relatively high percentage of patients requiring revision surgery underscores the importance of thorough preoperative assessment and accurate fracture reduction to minimize the risk of residual deformities.
